# PET/CT imaging for tumour response assessment to immunotherapy: current status and future directions

**DOI:** 10.1186/s41747-020-00190-1

**Published:** 2020-11-17

**Authors:** Marcus Unterrainer, Michael Ruzicka, Matthias P. Fabritius, Lena M. Mittlmeier, Michael Winkelmann, Johannes Rübenthaler, Matthias Brendel, Marion Subklewe, Michael von Bergwelt-Baildon, Jens Ricke, Wolfgang G. Kunz, Clemens C. Cyran

**Affiliations:** 1 Department of Radiology, University Hospital, LMU Munich, Marchioninistr. 15, 81377 Munich, Germany; 2 Department of Medicine III, University Hospital, LMU Munich, Munich, Germany; 3 Department of Nuclear Medicine, University Hospital, LMU Munich, Munich, Germany; 4 DIE RADIOLOGIE, Munich, Germany

**Keywords:** Antigens (neoplasm), Fluorodeoxyglucose F18, Immunotherapy, Positron emission tomography computed tomography, Receptors (chimeric antigen)

## Abstract

Recent immunotherapeutic approaches have evolved as powerful treatment options with high anti-tumour responses involving the patient’s own immune system. Passive immunotherapy applies agents that enhance existing anti-tumour responses, such as antibodies against immune checkpoints. Active immunotherapy uses agents that direct the immune system to attack tumour cells by targeting tumour antigens. Active cellular-based therapies are on the rise, most notably chimeric antigen receptor T cell therapy, which redirects patient-derived T cells against tumour antigens. Approved treatments are available for a variety of solid malignancies including melanoma, lung cancer and haematologic diseases. These novel immune-related therapeutic approaches can be accompanied by new patterns of response and progression and immune-related side-effects that challenge established imaging-based response assessment criteria, such as Response Evaluation Criteria in Solid tumours (RECIST) 1.1. Hence, new criteria have been developed. Beyond morphological information of computed tomography (CT) and magnetic resonance imaging, positron emission tomography (PET) emerges as a comprehensive imaging modality by assessing (patho-)physiological processes such as glucose metabolism, which enables more comprehensive response assessment in oncological patients. We review the current concepts of response assessment to immunotherapy with particular emphasis on hybrid imaging with ^18^F-FDG-PET/CT and aims at describing future trends of immunotherapy and additional aspects of molecular imaging within the field of immunotherapy.

## Key points

- Novel response criteria are incorporating positron emission tomography (PET) imaging to assess immunotherapy efficacy.
- PET-based response criteria refine the assessment of response to immunotherapy.
- PET can assist in detecting immune-related side effects.
- Novel PET-ligands targeting molecules in immune-related pathways are under development.

## Background

Recent immunotherapeutic approaches have emerged as powerful treatment options with high anti-tumour responses. These effects can be achieved by redirecting, stimulating, or genetically reprogramming the patient’s own immune system to target cancer cells. Passive immunotherapy is the most frequent form of immunotherapy, involving agents that enhance existing anti-tumour responses, such as antibodies against immune checkpoints such as cytotoxic T-lymphocyte-associated protein 4 (CTLA-4), programmed cell death protein (1 PD-1), and programmed death-ligand 1 (PD-L1). Active immunotherapy uses agents that direct the immune system to attack tumour cells by targeting tumour antigens (*e.g.*, vaccines such as Bacillus Calmette–Guérin in bladder cancer). Active cellular-based therapies are on the rise, most notably chimeric antigen receptor (CAR) T cell therapy, which redirects patient-derived T cells against tumour antigens [1–3].

These approaches are accompanied by novel patterns of response and progression, as clinical phenomena such as pseudoprogression or hyperprogression occur [4]; moreover, new aspects and manifestations of (immune-related) side-effects to systemic treatments can be observed [5]. Beyond these clinical features, current immunotherapeutic approaches do also challenge previously established imaging approaches based on computed tomography (CT) and magnetic resonance imaging. Beyond the assessment of the mere morphological extent, positron emission tomography (PET) imaging has emerged as comprehensive imaging modality by assessing (patho-)physiological processes and their changes to particular systemic treatments. Hence, combined hybrid imaging can highly influence the initial staging and the further clinical patient management in a high proportion of patients compared to morphological imaging only [6], as is currently for the clinical management of Hodgkin lymphoma [7].

This narrative work reviews the current concepts of response assessment to immunotherapy with a particular emphasis on combined hybrid imaging using ^18^F-FDG PET/CT and aims at describing future trends of immunotherapy and additional aspects of molecular imaging within the field of immunotherapy.

## Immunotherapy: the state of the art

The idea of utilising immune cells to eradicate malignant disease dates back to 1970, when Buckner et al. [8] reported the first successful allogeneic bone marrow transplantation in a patient suffering from leukaemia. This technique grew to become an indispensable means of treatment for many forms of haematologic malignancies. In contrast, recent immunotherapeutic approaches aim to achieve anti-tumour responses by redirecting, stimulating, or genetically reprogramming the patient’s own immune system to target cancer cells. These strategies include antibody-based treatments, immune checkpoint inhibitors, and chimeric antigen receptor (CAR)-T cells as most prominent examples.

Monoclonal antibodies offer the opportunity to therapeutically target specific tumour-associated antigens. By opsonisation, they enable effector cells such as natural killer cells [9], phagocytes [10, 11], and the complement system [12] to kill the respective target cells. Rituximab, targeting the cluster of differentiation (CD) 20 protein, is the most common agent and became essential for clinical routine since its approval in 1998 by the European Medicines Agency (EMA) for the treatment of haematologic B cell malignancies.

### Checkpoint inhibitors

Checkpoint inhibitors count among the most ground-breaking therapeutic approaches to have been translated into clinical use. The discovery of their underlying mode of action has been awarded with the Nobel Prize in Physiology or Medicine in 2018. The programmed cell death 1 (PD-1) protein, its corresponding programmed death-ligand 1 (PD-L1) and cytotoxic T lymphocyte-associated protein 4 (CTLA-4) represent immune checkpoints that are targeted in clinical practice. PD-L1 is often overexpressed in tumour cells and interacts with the membrane bound PD-1 on T cells, thus inhibiting T cell responses [1]. CTLA-4 is located intracellularly in resting T cells and translocates to the cell surface upon engagement of the T cell receptor, inhibiting activation of the T cell by competing for essential costimulatory binding sites and via inhibitory signalling [13]. Several checkpoint inhibitors have been approved for clinical use by the EMA and the United Stated Food and Drug Administration, first being Ipilimumab (anti-CTLA-4) in 2011, followed by others such as Nivolumab and Pembrolizumab (anti-PD-1).

### Bispecific T cell engagers and CAR-T cell therapy

Antibody therapies have advanced over time and recently the concept of bispecific antibodies or “bispecific T cell engagers” came into the spotlight, as the first bispecific T cell engager, Blinatumomab, was approved by the EMA in 2015 for use in relapsed or refractory B-precursor acute lymphoblastic leukaemia. This antibody is composed of two single-chain variable fragments targeting CD19 or CD3, respectively [14]. The bispecific nature of the antibody allows to bring tumour and immune effector cells into close proximity, facilitating the induction of immune cell mediated apoptosis [15].

CAR-T cells constitute the latest breakthrough in clinical immuno-oncology, with the two CAR-T cell products *Yescarta* (axicabtagene ciloleucel) and *Kymriah* (tisagenlecleucel) receiving approval by the EMA in August 2018, shortly after approval in the USA. The treatment is based on genetic modification of patient-derived T cells, obtained by leukapheresis, followed by their reinfusion into the patient. The cells are equipped with artificial CARs, which are composed of an antibody-derived single-chain variable fragments, a transmembrane and a signalling domain [16]. The single-chain variable fragment allows recognition of surface bound tumour-associated antigens such as CD19, whereas physiological T cell receptors are restricted to recognition of antigen fragments presented via the major histocompatibility complex [2]. Upon antigen binding, the CAR induces activation, proliferation of and cytokine release by the T cells, followed by cytotoxic activity targeted against the respective cancer cells. So far, the EMA approval covers therapeutic use in relapsed or refractory diffuse large B cell lymphoma, primary mediastinal large B cell lymphoma (Yescarta) and B cell Acute lymphoblastic leukaemia (Kymriah).

The consequences of CAR-T cell approval for clinical reality are remarkable, as the overall response rates for Kymriah during pivotal studies in patients suffering from relapsed or refractory diffuse large B cell lymphoma reached 52%, with 40% of the patients experiencing a complete response (CR), of whom 79% would remain relapse-free after 12 months of follow-up [17]. Yescarta demonstrated comparable efficacy with an objective response rate of 82% and a CR rate of 40% after a median follow-up time of 15.4 months [18, 19]. The endpoint for both pivotal studies had been set at best overall response in more than 20% of patients, a value based on data of historical studies. The significance at which this endpoint was met implies the overwhelming impact the approval had on the perspective of lymphoma patients relapsing from initial treatment regimens.

### Conventional imaging: pseudoprogression and hyperprogression

Standardised assessment of change in tumour burden is essential in the evaluation of therapies in cancer patients. Most clinical trials use tumour shrinkage (objective response) or development of progressive disease (PD) as endpoints and continuation or modification of therapy regimens depend on it. The Response Evaluation Criteria in Solid tumours (RECIST) guidelines were introduced by an international working group in 2000 [24] and revised in 2009 as RECIST 1.1 [20, 25]. RECIST is primarily based on the use of computed tomography (CT) and magnetic resonance imaging (Table 1). These criteria have been successfully validated in many studies, and today, most clinical trials on cancer therapies use them to evaluate objective tumour response even though, compared with chemotherapeutic drugs, tumours respond differently to newer drugs with other target mechanisms such as immunotherapeutics. Atypical response patterns such as *pseudoprogression* (where the tumour burden increases initially due to an increase in lesion size and/or occurrence of newly detectable tumour lesions with subsequent decrease in tumour burden) may lead to incorrect determination of the response status using RECIST [26].

**Table 1:** Overview of criteria for anatomical response evaluation to immunotherapy

Criteria (year) [reference]	Categories			
	Complete response	Partial response	Stable disease	Progressive disease
RECIST 1.1 (2009) [20]	• Disappearance of all TL/NTL • Nodal SAD < 1.0 cm • No new lesions	• ≥ 30% decrease of tumour burden relative to baseline • No new lesions	• Neither CR, PR, nor PD	• ≥ 20% increase of tumour burden relative to baseline • Or progression of NTL • Or new lesion(s)
irRC (2009) [21]	• Disappearance of all lesions (measurable or not) • No new lesions • Confirmation by consecutive CSI control in ≥ 4 weeks	• ≥ 50% decrease of tumour burden relative to baseline • Confirmation by consecutive CSI control in ≥ 4 weeks	• Neither CR, PR, nor PD	• ≥ 25% increase of tumour burden relative to nadir • New lesions added to tumour burden • Confirmation by consecutive CSI control in ≥ 4 weeks
irRECIST (2013) [22]	• Disappearance of all TL/NTL • Nodal SAD < 1.0 cm • No new lesions	• ≥ 30% decrease of tumour burden relative to baseline • No new lesions	• Neither CR, PR, nor PD	• ≥ 20% increase of tumour burden • And ≥ 5 mm absolute increase in total measured tumour burden relative to nadir (*i.e.*, minimum recorded tumour burden). • Confirmation of progression in ≥ 4 weeks after suspected PD
iRECIST (2017) [23]	• Disappearance of all TL/NTL • Nodal SAD < 1.0 cm • No new lesions	• Decrease of tumour burden > 30% relative to baseline • No new lesions	• Neither CR, PR, nor PD	iUPD: PD RECIST 1.1 iCPD: • Confirmation 4–8 weeks later • Any further size increase in TL sum > 5 mm • Any progression of NTL • Any further size increase of the sum of new TL > 5 mm • Appearance of another new lesion

*CSI* Cross-sectional imaging, *iCPD* Immune confirmed progressive disease, *iUPD* Immune unconfirmed progressive disease, *NTL* Non-target lesions, *SAD* Short-axis diameter, *TL* Target lesions

Since tumour growth or newly detectable tumour lesions are generally classified as PD based on RECIST, pseudoprogression is not diagnosed correctly and may result in an erroneous discontinuation of treatment or an unjustified exclusion of patients from clinical trials. To address this, the RECIST working group developed a modified guideline for response assessment to immunotherapy in 2017, called Immune RECIST (iRECIST) [23]. It is based on RECIST 1.1 guidelines and essentially has a new category of *immune unconfirmed progression disease* that requires to be confirmed by an additional, early follow-up scan within six to eight weeks. Immune unconfirmed progression disease should be considered carefully, as an increase in tumour size is still more likely to be true progression rather than pseudoprogression. The frequency of pseudoprogression varies between different tumour entities and is most frequently observed in melanoma patients (up to 13%) [21, 27]. Notably, in iRECIST, the target response drives the timepoint response after patients had immune unconfirmed progressive disease. For example, new lesions develop on follow-up 1 and persist or have not fully disappeared in follow-up 2. Yet, the target lesion sum on follow-up 2 has then regressed to a partial response (PR) level compared to baseline (in the absence of any other manifestations of PD). This is considered overall immune partial response, not continued immune unconfirmed PD.

Another atypical response pattern related to immunotherapy is hyperprogression, a term with various definitions, meaning a pronounced acceleration of tumour growth [28–30]. If hyperprogression is suspected, treatment must be interrupted immediately, even though robust biomarkers are still pending. Beyond iRECIST, several other refined response criteria using morphological information were developed (see Table 1).

### PET/CT imaging of immunotherapy

#### ^18^F-FDG PET-based response assessment criteria

In 1999, the European Organization for Research and Treatment of Cancer’ (EORTC) first introduced PET-based, metabolic information in specified criteria for the response assessment of oncological diseases in general [31]. Of note, those were also the first PET-based criteria to be applied for monitoring of immunotherapy [32]. These EORTC criteria were then superseded by the PET Response Criteria in Solid tumours (PERCIST) published by Wahl et al. in 2009 [33]. Despite rather comparable classifications, PERCIST introduced the SUL—which is the standardised uptake value (SUV) corrected for the lean body mass—as an imaging parameter and made a tumour SUL 1.5-fold higher than the SUL of the non-affected liver a prerequisite for an evaluable lesion. Moreover, the SUL_peak_ is assessed within a spherical volume of interest in the site of the most metabolically active tumour manifestation.

In 2017, Cho et al. [34] prospectively compared different response criteria in a small set of patients undergoing immunotherapy in order to evaluate an optimised complementary fit between morphological and metabolic response parameters. The best combination of the assessed parameters were then transformed in new criteria and named *PET/CT Criteria for Early Prediction of Response to Immune Checkpoint Inhibitor Therapy* (PECRIT) [34]. Additional response criteria were suggested by the Heidelberg group by introducing *PET Response Evaluation Criteria for Immunotherapy* (PERCIMT), which moreover take into account the clinical relevance of the absolute amount of new lesions during immunotherapy [35, 36]. For an overview, please see Table 2.

**Table 2:** Overview metabolic and combined response evaluation to immunotherapy

Criteria (year) [reference]	Modality	Categories			
		Complete response	Partial response	Stable disease	Progressive disease
EORTC (1999) [31]	PET	• Reduction of ^18^F-FDG uptake to background levels	• ≥ 15% reduction of ^18^F-FDG uptake	• Neither CR, PR, nor PD	• ≥ 25% increase in ^18^F-FDG uptake
PERCIST (2009) [33]	PET	• Reduction of ^18^F-FDG uptake to the level of background blood pool	• ≥ 30% reduction in SUL peak • Minimum of 0.8 SUL units of measurable lesions	• Neither CR, PR, nor PD	• > 30% increase in SUL peak • Minimum of 0.8 SUL units of measurable lesions
PECRIT (2017) [34]	PET/CT	• Disappearance of all metabolically active tumours and TL • SAD reduction target lymph nodes < 10 mm • No new lesions	• ≥ 30% reduction in SUL peak • ≥ 30% decrease in TL diameter sum	• Neither CR, PR, nor PD	• > 30% increase in SUL peak • Or new metabolically active lesion • ≥ 20% increase in target lesion diameter (minimum 5 mm) • Or new lesions
PERCIMT (2018) [35]	PET/CT	• Complete resolution of all ^18^F-FDG-avid lesions • No new FDG avid lesions	• Complete resolution of some ^18^F-FDG-avid lesions • No new ^18^F-FDG-avid lesions	• Neither CR, PR, nor PD	• ≥ 4 new lesions with ≤ 10 mm functional diameter • Or three or more new lesions with > 10 mm functional diameter • Or two or more new lesions with > 15 mm functional diameter

*CR* Complete response*, CT* Computed tomography, *FDG* Fluorodeoxyglucose, *PD* Progressive disease*, PET* Positron emission tomography, *PR* Partial response, *SAD* Short-axis diameter, *SUL* SUV corrected for lean body mass, *TL* Target lesions

When dealing with lymphomas, specific criteria for response assessment were established. In 1999, the first standardised response criteria for lymphoma were introduced [37]. However, the issue of residual morphological masses remained unsolved. Hence, in 2007, Cheson criteria incorporated PET imaging for ^18^F-FDG-avid lymphomas [38]. Based on the *First International Workshop on PET in Lymphoma* in Deauville, France, a newly established 5-point scale (*i.e.*, the Deauville score) relative to blood-pool and liver activity (see Table 3) was introduced [39] and, as a consequence, was incorporated in the subsequent Lugano criteria in 2014 [40], which succeeded the Cheson criteria. In general, a Deauville score 1–3 during therapy is considered as CR, whereas a score 4–5 at the termination of treatment is considered a non-response.

**Table 3:** Five-point Deauville score system

Score	Metabolic activity of lymphoma
**1**	No ^18^F-FDG-uptake above background activity
**2**	^18^F-FDG-uptake ≤ mediastinal blood pool activity
**3**	^18^F-FDG-uptake between mediastinal blood pool and liver activity
**4**	^18^F-FDG-uptake moderately higher than liver activity
**5**	^18^F-FDG-uptake markedly higher than liver activity/new lesion(s)
**X**	New areas of ^18^F-FDG-uptake unlikely related to lymphoma

*FDG* Fluorodeoxyglucose

In the light of immunotherapy, modified Lugano criteria (lymphoma response to immunomodulatory therapy criteria (LYRIC)) were proposed in 2016 to account for features specific for immunotherapy [41]. Here, the category *indeterminate response* (IR) was introduced when an increase of tumour burden, new lesions, or an increase of ^18^F-FDG-avidity is observed, leading to a consequent follow-up imaging study within twelve weeks in order to rule out or confirm PD or pseudo-progression [41].

Most recently, in 2017, *Response Evaluation Criteria in Lymphoma* (RECIL) were established by an international working group [42]. RECIL aimed at homogenising response assessing in trials by modifying response criteria. Within this process, the role of ^18^F-FDG PET was reduced in favour of a more pronounced impact of CT-based changes, considering the potential alteration of glucose metabolism by immunomodulatory drugs that may obscure the tumour ^18^F-FDG-avidity [42]. In Table 4, an overview of response criteria for lymphoma is provided.

**Table 4:** Overview of response criteria for lymphoma

Criteria (year) [reference]	Categories			
	Complete response	Partial response	Stable disease	Progressive disease
Lugano (2014) [40]	• CT: reduction of lesions to normal size • PET: normalised ^18^F-FDG-uptake (DS 1–3)	• CT: ≥ 50% reduction in SPD of up to 6 lesions • PET: reduced ^18^F-FDG- uptake (DS 4 or 5)	• CT: neither sufficient change for PD nor PR • PET: unchanged ^18^F-FDG-uptake (DS 4 or 5)	• CT: ≥ 50% increase in SPD of lesions • New lesion(s) • PET: increased ^18^F-FDG-uptake (DS 4 or 5) or new ^18^F-FDG-avid lesions
LYRIC (2016) [41]	• Same as Lugano	• Same as Lugano	• Same as Lugano	Adapted from Lugano to indeterminate response (IR) categories: • IR_1_: ≥ 50% increase in SPD in 12 weeks without clinical deterioration • IR_2_: < 50% increase in SPD with new lesion(s), or ≥ 50% increase in SPD of a lesion or set of lesions at any time during treatment • IR_3_: increase in ^18^F-FDG-uptake without increase in lesion size meeting criteria for PD
RECIL (2017) [42]	• CT: complete disappearance of all TL and all nodes with LD < 10 mm • PET: normalised ^18^F-FDG-uptake (DS 1–3)	Partial response • CT: ≥ 30% decrease in SLD of TL, but no CR • PET: DS 4 or 5	• CT: < 10% decrease or ≤ 20% increase SLD of TL • PET: any DS	• CT: > 20% increase in SLD of TL • For small lymph nodes < 15 mm after therapy, a minimum absolute increase of 5 mm and the LD > 15 mm • New lesion(s) • PET: any DS
		Minor response • Same as PR yet only ≥ 10% and < 30% SLD decrease		

*CT* Computed tomography, *DS* Deauville score, *FDG* Fluorodeoxyglucose, *IR* Indeterminate response, *LD* Long diameter, *PD* Progressive disease*, PET* Positron emission tomography, *PR* Partial response, *SLD* Sum of longest diameters, *SPD* Sum of perpendicular diameters, *TL* Target lesions

### Response assessment to immunotherapy with ^18^F-FDG PET

PET imaging was initially used for immunotherapy monitoring in patients with solid tumours. Given the early and successful implementation of immunotherapy within the clinical workup of melanoma patients, the first PET-based response assessment using EORTC criteria was applied in melanoma patients [32]. Already at this early stage, the appearance of new lesions was not linked to progressive disease *per se* leading to potential misclassifications. Residual metabolic activity on ^18^F-FDG PET (similarly to the Deauville score assessment) in melanoma patients treated with anti-PD-1 agents was also associated with residual vital tumour masses. *Vice versa*, a loss of ^18^F-FDG-avidity despite remaining morphological masses was associated with improved outcome; however, remaining residual ^18^F-FDG-avidity despite clinical response was also observed, possibly due to immune infiltrates [43].

In a cohort of melanoma patients undergoing immunotherapy, several morphological and functional response assessment criteria were applied, but only a limited agreement among the applied criteria in terms of outcome prediction was observed. Hence, the combination of parameters best suitable for prediction was established (PECRIT criteria) [34]. When dealing with immunotherapy using ipilimumab in melanoma, the Heidelberg group demonstrated that changes of SUV-based parameters in the disease course do not predict the individual outcome, whereas the number of new lesions and their extent during therapy was predictive for clinical outcome and allowed proper stratification [35] (see also Table 4).

As a consequence, PERCIMT criteria were also used for interim evaluation in melanoma patients undergoing immunotherapy and compared to EORTC criteria by stratifying patients with metabolic benefit (*i.e.*, CR, PR, or SD) and those without (*i.e.*, PD). Again, agreement of PERCIMT and EORTC was limited; although PERCIMT showed a significantly higher sensitivity for the prediction of clinical benefit than EORTC, both criteria were equally able to predict the absence of clinical benefit [36]. Hence, a recent position paper by the European Association of Nuclear Medicine critically discussed the added value of the PERCIMT criteria. Firstly, study conclusions were based on 41 patients only and secondly, EORTC criteria showed even slightly higher diagnostic performance for the detection of a missing clinical benefit compared to PERCIMT, without reaching the level of significance [35].

Beyond melanoma, a few studies have addressed the value of ^18^F-FDG PET imaging of non-small cell lung carcinoma (NSCLC) patients undergoing immunotherapy. Here, ^18^F-FDG changes in terms of PERCIST criteria (compared to RECIST 1.1) were highly predictive for treatment efficacy in NSCLC patients undergoing nivolumab therapy even at an early stage of 1 month after treatment initiation and was shown to be an independent prognostic factor at multivariate analysis [44]. Also, response on ^18^F-FDG PET (using EORTC criteria) in NSCLC patients undergoing atezolizumab therapy 6 weeks after initiation were predictive for the further morphological disease course on CT. Moreover, even patients with pseudoprogression could be identified by using ^18^F-FDG PET [45]. In addition, follow-up ^18^F-FDG PET imaging in patients classified as PD on PERCIST criteria was able to identify patients with pseudoprogression and immune dissociated-response in more than half of patients previously classified as PD. Importantly, improved clinical outcome was observed in these patients [46].

When dealing with haematologic malignancies, first reports of anti-PD1-therapy in Hodgkin lymphoma (HL) were published already in 2014 [47], where a combination of CT and PET/CT imaging was used in order to assess response to immunotherapy. The KEYNOTE-013 trial [48] applied the Cheson 2007 criteria [38]. These and their updated 2014 version, the Lugano criteria [40], were subsequently applied in several trials evaluating immunotherapy in HL [49–53], partly also in comparison to LYRIC criteria [54, 55].

**Fig. 1:**
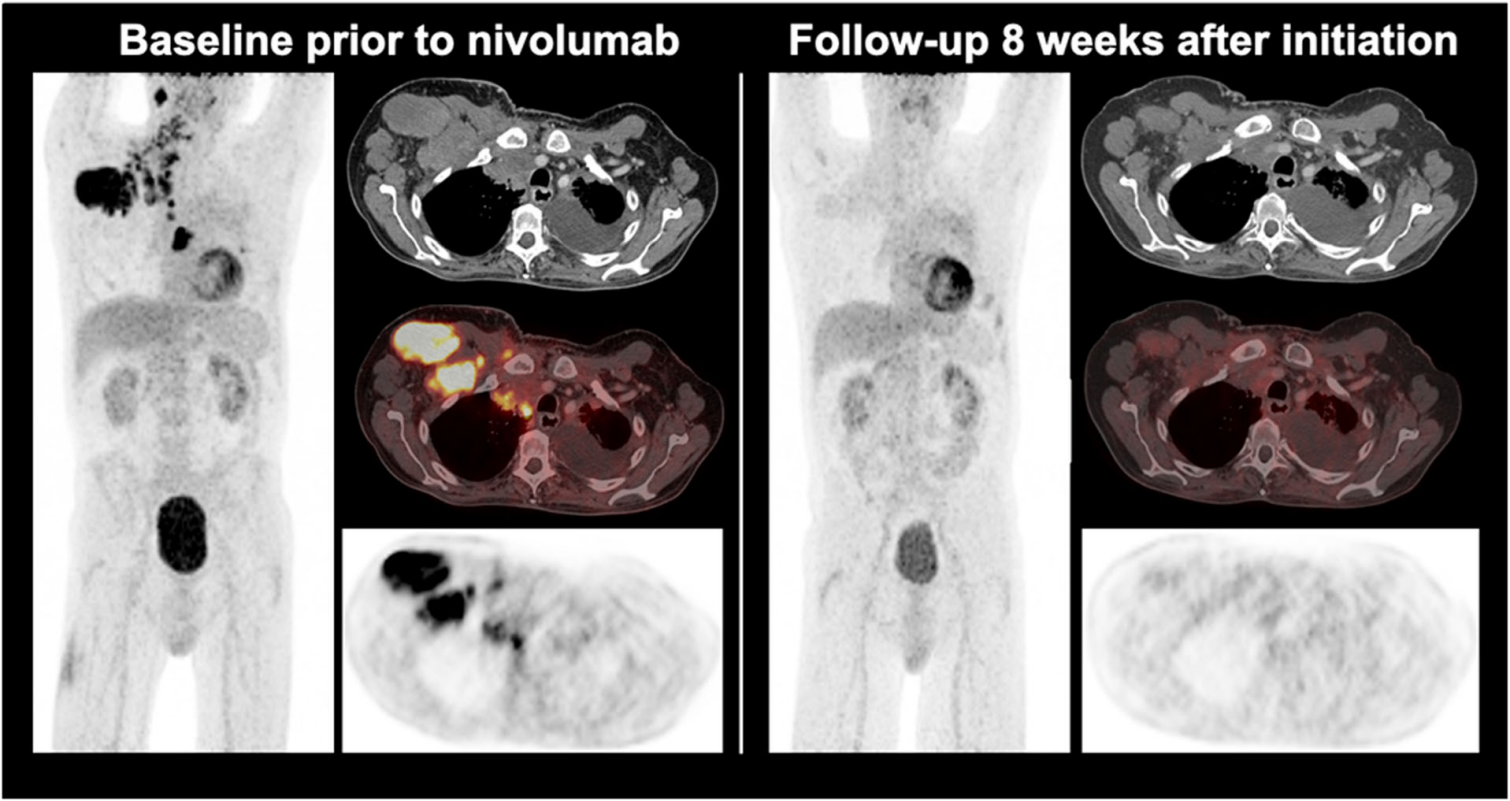
A Hodgkin lymphoma patient with metabolically active tumour manifestations prior to the initiation of immunotherapy with nivolumab. A complete metabolic response already 8 weeks after immunotherapy initiation despite remaining morphological masses on CT was observed

Initially, the metabolic changes over time in patients with relapsed or refractory HL undergoing anti-PD-1-treatment were described by Dercle et al. [56]. Subsequently, the same group demonstrated that a decrease of ^18^F-FDG-avidity in tumour and spleen as well as the general ^18^F-FDG-avid tumour burden 3 months after initiation of anti-PD-1-treatment were associated with improved clinical outcome [55]. Consequently, CT-based response evaluation had to be reclassified when additionally applying PET criteria in 44% of HL patients undergoing nivolumab. Among these, the majority showed complete metabolic response in contrast to CT (Fig. 1) with improved clinical outcome [57]. In the setting of early treatment response of HL and anti-PD-1-treatment, both Lugano criteria and LYRIC performed equally with equivocal findings [58], a result that possibly relates to the rather rare occurrence of pseudoprogression in HL [55, 56, 59].

**Fig. 2:**
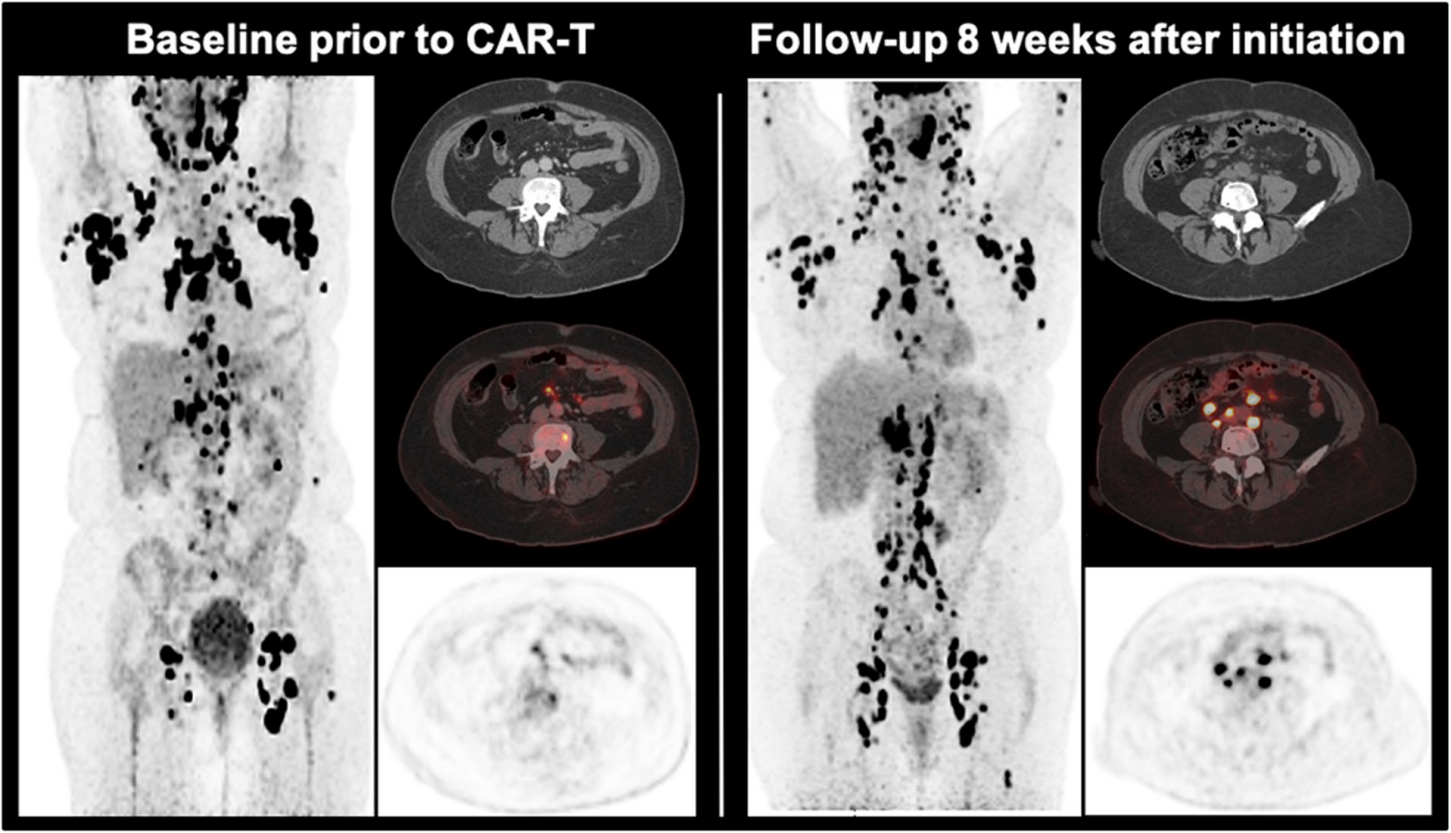
A patient example with pseudoprogression of diffuse large B cell lymphoma undergoing chimeric antigen receptor T (CAR-T) cell therapy. Eight weeks after reinfusion of CAR-T cells, numerous abdominal lymph nodes with highly increased metabolism occurred, but fully resolved in the further disease course without additional treatment

With regard to PET/CT imaging in CAR-T cell therapy, only limited data is available. Firstly, Shah et al. [60] demonstrated in a small set of diffuse large B cell lymphoma and follicular lymphoma that patients with complete remission of the metabolic tumour volume on ^18^F-FDG PET 4 weeks after CAR-T cell therapy showed a long-term remission over 2 years and patients with remaining activity had an early relapse. Secondly, Wang et al. [61] showed that a higher ^18^F-FDG-avid tumour burden prior to therapy was associated with more severe CAR-T cell therapy-related side effects. Interestingly, this study also demonstrated that the phenomenon of pseudoprogression and local immune activation can also occur in patients undergoing CAR-T cell therapy (Fig. 2). Of note, several trials are underway evaluating the particular contribution of PET imaging in the course of CAR-T cell therapy (*e.g.*, NCT03086954, NCT02476734).

So far, there is only limited literature dealing with the very exact clinical value of PET/CT imaging for the identification of pseudo-progression in patients undergoing immunotherapy. One study in melanoma patients suggests that the new appearance of ≥ 4 metabolically active lesions with a functional diameter < 1.0 cm or ≥ 3 lesions > 1.0 cm is associated with real progression rather than the occurrence of pseudoprogression [35]. Another study identified that true progression was associated with a larger increase of metabolic tumour volume than pseudoprogression at the time of first follow-up [62]. More recent data indicated that metabolic changes of primary and secondary lymphoid organs during the course of immunotherapy in melanoma patients are associated with therapy response [63]; hence, these changes of lymphoid organs such as the spleen could potentially be useful for the differentiation of pseudo-progression and real progression. These interesting findings should be explored in further studies to assess their diagnostic value for early identification of patients with pseudoprogression.

### Imaging of immune-related adverse events

During the application of immunotherapeutic agents, there is a reactivation of the immune system that not only has anti-tumour effects, but also might affect healthy tissue leading to new toxicity profiles that require a different management than the toxicity of chemotherapies [64, 65]. These new immune-related adverse events (irAE) present with a broad variety of symptoms and might affect a multitude of organs. Most commonly, the cutaneous, gastrointestinal and endocrine systems are affected. However, some differences and diverging patterns of clinical manifestations can be observed depending on the checkpoint inhibitor and immunotherapy subgroups [66]. Nonetheless, a rapid identification of irAEs can improve the clinical outcome, as most of these irAEs are treated with subsequent systemic immunosuppression [67, 68].

The occurrence of irAEs can also affect response assessment with PET, as inflammatory reactions accompany these irAEs consequently leading to an elevated ^18^F-FDG-avidity [69], which might lead to a misinterpretation of the respective PET study. However, a certain adaptation of ^18^F-FDG-avidity can be observed over time [70, 71]. Vice versa, this ^18^F-FDG-avidity of irAEs also enables an exact localisation and identification [72], which gains further importance in the light of the association of occurrence of irAEs and the effectiveness of immunotherapy in melanoma and NSCLC patients [5, 73].

**Fig. 3:**
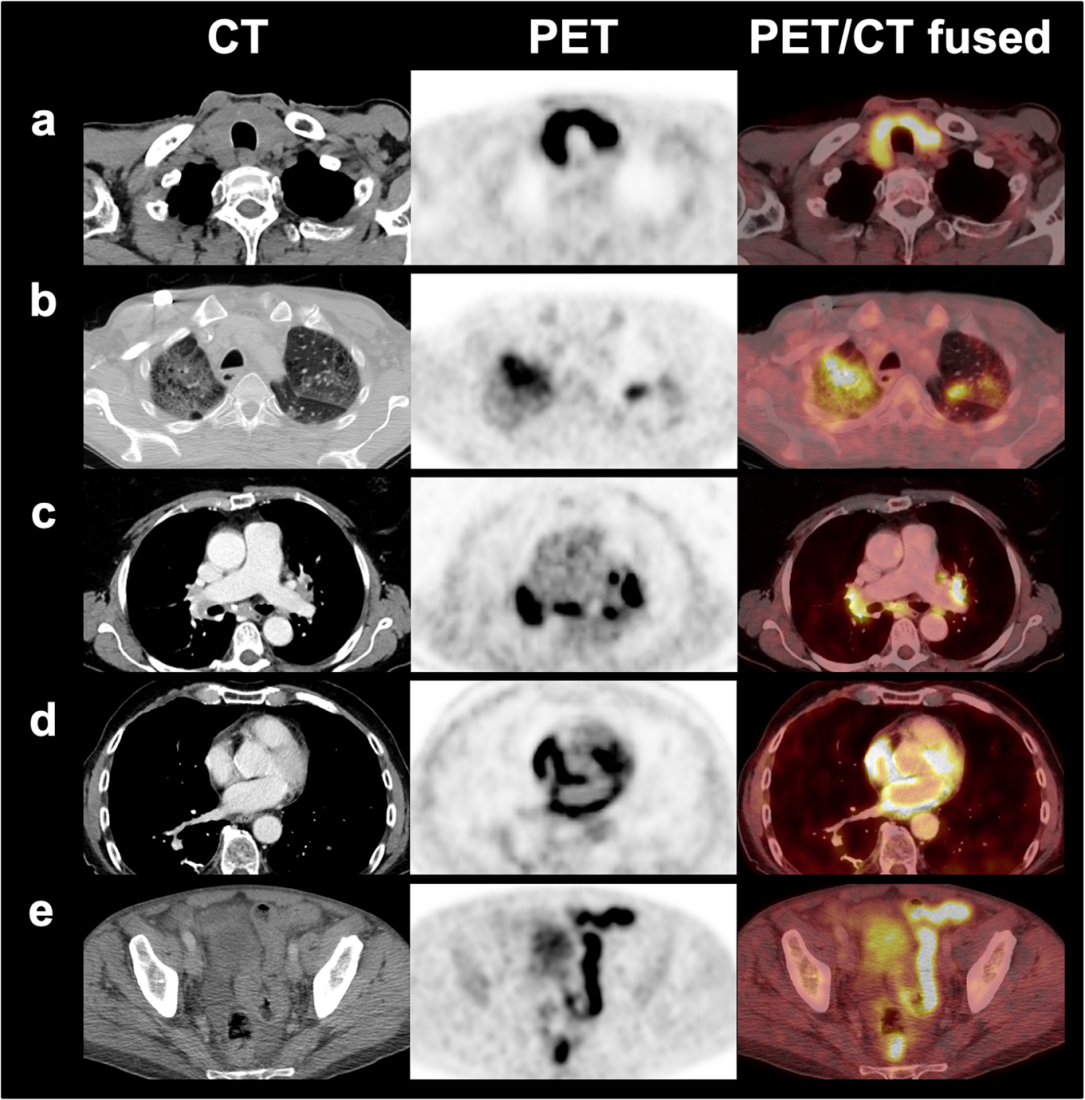
Examples of immune-related adverse events on positron emission tomography/computed tomography with intensely increased ^18^F-FDG uptake. **a** Thyroiditis. **b** Pneumonitis. **c** Sarcoid-like reaction. **d** Pericarditis. **e** Colitis

Recently, the report from the European Association of Nuclear Medicine symposium on immunotherapy stated that incidental findings related to irAEs should be reported. Although irAEs might not necessarily be associated with clinical symptoms, clinicians should be aware of their presence and ensure clinical monitoring, which, however, might lead to a clinical intervention. First signs of elevated immune activity can be seen as spleen enlargement and/or elevated uptake leading to an inversion of the liver-to-spleen uptake ratio. Also, reactive lymph nodes might be observed in the direct drainage of the tumour. However, these findings have to be compared to the respective baseline scan to assess their pathophysiological relevance, but also to safely relate these findings to the immunotherapy [74]. Moreover, the occurrence of immune-related *sarcoid-like reactions* consisting of lymphadenopathy and pulmonary granulomatosis with elevated glucose consumption have to be kept in mind [75]. This is also the most relevant irAE that may be misinterpreted as progression by mimicking newly developed mediastinal and hilar lymph node manifestations. The discordant course of other manifestations and the symmetry of these changes are helpful for the differentiation from malignant lesions (Fig. 3).

In CAR-T cell therapy, there is, however, another set of rather immediate adverse effects, such as cytokine release syndrome, CAR-T cell-related neurologic toxicities, and B cell aplasia, which are not directly detectable using ^18^F-FDG-PET [61, 76, 77]. Moreover, CAR-T cell-related adverse events occur even earlier than irAEs, even hours and days after the first application. In sum, there is only a small amount of literature describing late toxicities different from irAEs so far [78, 79]. Hence, more clinical experience and, in particular, literature evaluating the use of PET imaging in CAR-T cell therapy are needed.

## Future directions

### Novel treatments

Despite growing success, immunotherapy, adoptive cell therapy in particular, still face many challenges. As the scientific community remains in search for answers as to why significant fractions of patients remain nonresponsive to immunotherapy, new targets for cellular treatments are validated in a fast-growing number of clinical trials.

In the context of haematologic malignancies, these new approaches include CAR-T cells targeted against B cell maturation antigen in relapsed or refractory multiple myeloma [80, 81] and CAR-T cells targeted against CD22 in acute lymphoblastic leukaemia, last of which managed to achieve stunning CR rates of over 80% in patients treated at the highest-dose level in a phase I trial [3, 82]. Further, CAR-T cells are evaluated in multiple phase I and II trials for use in solid malignancies such as mesothelioma, metastatic pancreatic, gastric and prostate cancers, glioblastoma, sarcoma, and others [83].

Another emerging concept in cellular immunotherapy is universal or adapter CAR-T cells. The single-chain variable fragments of these CARs are designed to recognize antigens which are physiologically not present on the surface of tumour or healthy cells [84]. Application of tumour-specific ligands linked to such antigens allows them to serve as an adapter between the universal CAR and the respective tumour cell. This enables targeting of a broad variety of tumour antigens simultaneously or sequentially and without the need to engineer CAR-T cells for every single tumour under consideration, while at the same time providing better control of CAR-T cell activity [85, 86].

Taking the idea of universal immune cells further, recent reports demonstrate the potential of CAR-transduced natural killer cells to combat lymphoma in a combined phase I and II trial [87]. Interestingly, natural killer cells do not mediate graft-*versu*s-host-disease due to their lack of endogenous T cell receptors, allowing human leukocyte antigen mismatched transfusions [3]. These early data suggest that an off-the-shelf CAR product may be within reach, which eventually will be necessary to enable broad availability and affordability.

### Novel ligands for nuclear imaging

Beyond morphological and glucose-based imaging, new molecular radiotracers arise that directly target the key molecules of immune checkpoint pathways and immune responses [88, 89]. Anti-PD-1 antibodies can be labeled with ^89^Zr or ^64^Cu and are suitable for in vivo imaging PD-1-expressing tumour-infiltrating lymphocytes [89], which might be an interesting approach for the non-invasive visualisation and quantification of PD-1-expression, as immunohistochemical analyses are limited by the heterogeneous tissue expression on biopsies or single tissue specimen [90].

First studies were already performed in humans. Niemeijer et al. [91] published a study using radiolabeled anti-PD-1 monoclonal antibody ^89^Zr-nivolumab in patients with advanced NSCLC and showed a significant ^89^Zr-nivolumab tumour uptake, that was higher in patients with immunohistochemically proven PD-1 positive tumour-infiltrating immune cells as compared with PD-1 negative tumours. Interestingly, PD-(L)1 PET-CT demonstrated highly heterogeneous tumour uptake inter-individually, but also intra-individually with divergent uptake between different tumour lesions [90, 92]. Moreover, high uptake on pretreatment ^89^Zr-atezolizumab (an antibody to PD-L1) PET showed stronger correlation with the clinical outcome than immunohistochemistry- or ribonucleic acid-sequencing-based biomarkers in patients subsequently undergoing PD-L1-targeted therapies.

Several trials in humans aimed at establishing novel immuno-PET ligands in a broad range of cancer entities such as ^89^Zr-avelumab PET in NSCLC (NCT03514719, PINNACLE trial) or ^89^Zr-durvalumab in head-and-neck squamous cell cancer (NCT03829007, PINCH trial). Also, dual imaging approaches with ^18^F-FDG PET are on the way, for example combining ^18^F-FDG PET and ^18^F-PD-L1 PET in oral cavity squamous cell cancer (NCT03843515, NeoNivo trial)

Beyond imaging PD-L1 using PET, several interesting biomarkers were introduced to molecular imaging in preclinical settings such as interferon-γ immuno-PET (^89^Zr-anti-IFN-γ) that allows imaging of activated lymphocytes inside tumour lesions [93]. Another interesting target is represented by the protease granzyme B (GZP). It is secreted by cytotoxic CD8+ during immune-induced, caspase-dependent apoptosis. Targeting imaging with ^68^Ga-NOTA-GZP allowed prediction of response to immunotherapy with high accuracy in preclinical models [94]. Beyond the scope of PET imaging, also promising molecular structures can be targeted using single-photon emission tomography ligands. Among them, a very encouraging perspective is offered by ^99m^Tc-labeled interleukin-2 (^99m^Tc-HYNIC-IL2), which demonstrated feasibility for visualisation and quantification of tumour infiltrating lymphocytes in a small set of melanoma patients undergoing immunotherapy, so providing a potential non-invasive tool for the differentiation between progression and pseudoprogression [95].

These promising efforts in both preclinical and clinical setting underline the further investigation of immuno-PET and the comprehensive translation into clinical imaging to further improve pretreatment patient selection, response assessment and clinical management. Moreover, artificial intelligence algorithms are increasingly used to evaluate treatment response by evaluating image-derived biomarkers [96, 97], which can also incorporate PET-derived information. Future trends also head towards *integrated diagnostics’*, *i.e.*, combining multiparametric diagnostic data from imaging, pathology, molecular genetics, and liquid biopsies, with final aim of therapy guidance.

## Data Availability

Data sharing is not applicable to this article as no datasets were generated or analysed during the current study.

## Abbreviations

CAR: Chimeric antigen receptor
CD: Cluster of differentiation
CR: Complete response
CT: Computed tomography
CTLA-4: Cytotoxic T-lymphocyte-associated protein 4
EMA: European Medicines Agency
EORTC: European Organization for Research and Treatment of Cancer
FDG: Fluorodeoxyglucose
GZP: Protease granzyme B
HL: Hodgkin lymphoma
irAE: Immune-related adverse events
iRECIST: Immune Response Evaluation Criteria in Solid tumours
irRC: Immune-related response criteria
LYRIC: Lymphoma response to immunomodulatory therapy criteria
NSCLC: Non-small cell lung cancer
PD: Progressive disease
PD-1: Programmed cell death protein 1
PD-L1: Programmed death-ligand 1
PECRIT: PET/CT criteria for early prediction of response to immune checkpoint inhibitor therapy
PERCIMT: PET Response Evaluation Criteria for Immunotherapy
PERCIST: PET Response Criteria in Solid tumours
PET: Positron emission tomography
PR: Partial response
RECIL: Response evaluation criteria in lymphoma
RECIST: Response Evaluation Criteria in Solid tumours
SD: Stable disease
SUL: Standardised uptake value corrected for the lean body mass
SUV: Standardised uptake value
